# Personal factors influence use of cervical cancer screening services: epidemiological survey and linked administrative data address the limitations of previous research

**DOI:** 10.1186/1472-6963-12-34

**Published:** 2012-02-14

**Authors:** Sarah C Olesen, Peter Butterworth, Patricia Jacomb, Robert J Tait

**Affiliations:** 1 Psychiatric Epidemiology and Social Issues Unit, Centre for Research on Ageing, Health and Wellbeing, The Australian National University, Building 63 Eggleston Road, Acton, ACT, Australia 0200; 2 Centre for Research on Ageing, Health and Wellbeing, The Australian National University, Building 63 Eggleston Road, Acton, ACT, Australia 0200; 3 Centre for Mental Health Research, The Australian National University, Building 63 Eggleston Road, Acton, ACT, Australia 0200

## Abstract

**Background:**

National screening programs have reduced cervical cancer mortality; however participation in these programs varies according to women's personal and social characteristics. Research into these inequalities has been limited by reliance on self-reported service use data that is potentially biased, or administrative data that lacks personal detail. We address these limitations and extend existing research by examining rates and correlates of cervical screening in a large epidemiological survey with linked administrative data.

**Methods:**

The cross-sectional sample included 1685 women aged 44-48 and 64-68 years from the Australian Capital Territory and Queanbeyan, Australia. Relative risk was assessed by logistic regression models and summary Population Attributable Risk (PAR) was used to quantify the effect of inequalities on rates of cervical cancer screening.

**Results:**

Overall, 60.5% of women participated in screening over the two-year period recommended by Australian guidelines. Screening participation was associated with having children, moderate or high use of health services, employment, reported lifetime history of drug use, and better physical functioning. Conversely, rates of cervical screening were lower amongst women who were older, reliant on welfare, obese, current smokers, reported childhood sexual abuse, and those with anxiety symptoms. A summary PAR showed that effective targeting of women with readily observable risk-factors (no children, no partner, receiving income support payments, not working, obese, current smoker, anxiety, poor physical health, and low overall health service use) could potentially reduce overall non-participation in screening by 74%.

**Conclusions:**

This study illustrates a valuable method for investigating the personal determinants of health service use by combining representative survey data with linked administrative records. Reliable knowledge about the characteristics that predict uptake of cervical cancer screening services will inform targeted health promotion efforts.

## Background

Cancer of the cervix is the second most prevalent cancer among women worldwide. The greatest burden occurs in the developing world where the mortality rate ranges from 10 to 35 per 100 000 compared with two to four deaths per 100 000 in developed nations [1]. This difference is attributed to effective, national screening programs of cervical cytologic testing (the Papanicolaou test) to identify cell abnormalities that may indicate or precede cervical cancers [2,3].

Screening programs for cervical cancer typically target all asymptomatic women from soon after the onset of sexual activity to early old age. Australia's National Cervical Screening Program recommends screening every two years, one to two years after the onset of sexual activity, until the age 70 [4]. This guideline remains despite the introduction of the human papillomavirus (HPV) vaccine, which cannot prevent all types of HPV that can cause cervical cancer [5]. Despite the success of such programs, inequalities in the receipt of screening services continue amongst women at greater risk of poor health outcomes [6-8]. Consequently, researchers have attempted to identify women who are less likely to participate in screening to better target these services. These studies have reported that non participation is associated with being: single, sexually inactive, obese, a current smoker, uninsured, a non-metropolitan resident, having a low household income, lower level of education, current psychological distress, non-Caucasian background and attitudinal factors [8-17]. Collection of sociodemographic information to aid in identifying inequitable access has been advocated [8].

The accuracy of service use data alongside personal information is central to ability of such research to inform service delivery in a meaningful way. Former studies into the inequalities in cervical cancer screening and other preventative health services have used one of two approaches: (1) self-reported service use or (2) administrative records. Each of these approaches has strengths and weaknesses. Self-report methods have the advantage of being collected alongside the detailed personal information that is needed to investigate the determinants of screening participation and exclude people who are ineligible for the service (e.g., those who have had a hysterectomy; [18]). The accuracy of self-reported health service use is, however, subject to bias associated with social desirability [19], 'over-reporting' [18,20], inaccurate recall for the date of screening [20] and conflation with other health services [21] [though see: [22]]. There is typically only moderate concordance between self-report and medical records [19,23,24]. Importantly, *dis*cordance appears to be greater amongst women who are also less likely to receive cervical cancer screening [25-27]. Administrative data is considered accurate but lacks concurrent data on the personal characteristics that may drive or inhibit service use. The small number of studies that have combined personal and administrative data have been limited to unrepresentative samples such as primary health care settings that may exclude women who do not frequent health services [28].

The Personality and Total Health Through Life Study (PATH) uses a cross-sequential design to examine adult development with three representative, general community cohorts. These cohorts were aged 20-24, 40-44 and 60-64 years at baseline with follow-up every four years [29,30]. In addition to collecting comprehensive self-reported health and psychosocial data, over 90% of participants gave permission for their administrative health service records to be accessed.

This study addresses several methodological limitations of previous research into the determinants of cervical cancer screening and other preventative health services by combining the strengths of self-report and administrative approaches. We link self-reported psychosocial data with administrative records for screening service use. By doing so we are able to (1) estimate the rates of cervical cancer screening, (2) verify the characteristics thought to be associated with cervical cancer screening, (3) investigate whether these characteristics vary with women's age, and (4) quantify the effects of these inequalities with greater reliability.

## Methods

### Study design and setting

The PATH sample was derived via random selection from the electoral roll of Canberra (Australia's capital) and the neighbouring town of Queanbeyan. Registration on the electoral roll is compulsory for Australian adult citizens. Due to availability of linked administrative data, this analysis is restricted to wave two data for the two older cohorts. Wave two interviews were conducted in 2003-2004. The original response rates were 64.6% and 58.3%; the follow-up rates at wave two were 93.0% and 87.1%; and consent rates for administrative data linkage were 95.5% and 96.3%.

At each wave, participants complete self-report measures on health, lifestyle, psychological and social wellbeing and personality characteristics. Questionnaires are completed on a hand-held computer in the presence of an interviewer who also administers a battery of physical health tests. At the conclusion of the wave two interviews, participants were asked to consent to making available their administrative data on health services received over a two-year period, including records of participation in routine cervical cancer screening procedures. The Human Research Ethics Committee of The Australian National University approved the PATH study.

### Measures

#### Outcome

The dichotomous outcome variable for all analyses was receipt of a cervical cancer screening service according to administrative records ('0' represented no record of screening during the two-year period and '1' represented at least one screening test). Cervical screening is typically performed in a primary care setting by the individual's general practitioner (GP) or practice nurse and is a government-subsidised procedure in Australia. This service is recorded by the GP with Medicare Australia (the national health insurer).

#### Independent variables

Individual characteristics that are theorised, or previously demonstrated, to be associated with the uptake of preventative health screening services were considered. These included: age, marital and parent status, ethnicity, indicators of socio-economic position (financial hardship, welfare reliance, living in rental housing, employment status, childhood poverty, education), lifestyle/health risk behaviours (body mass index (BMI), alcohol use, current smoker status, lifetime use of marijuana, ecstasy or amphetamines, physical exercise), traumatic life experiences (lifetime history of rape and sexual molestation, childhood sexual and physical abuse; early sexual experience (< 15 years)), personality, and health status (self-rated general health, past hysterectomy, physical and mental health).

Participants were considered to be welfare reliant if their main source of income was reported to be a Government pension, allowance or benefit. Participants were asked whether they had experienced any of four instances of financial hardship (relating to a lack of basic goods or services due to inadequate resources) in the last year. These items were derived from the Australian Household Expenditure Survey [31,32]. Alcohol consumption was assessed using three items from the Alcohol Use Disorders Identification Test [33]. Based on the national guidelines [34] that were in place at the time, women consuming ≥ 14 and males ≥ 28 standard drinks per week were deemed hazardous/harmful users. A variable was constructed from items originally used in the Whitehall II study to assess participation in mild, moderate and vigorous exercise [35]. Personality was measured using the 36-item Eysenck Personality Questionnaire [36] (neuroticism and extroversion scales). Scores on these scales range from 0 to 12; higher scores indicate stronger traits. Mastery (or sense of control over one's life), was assessed using a 7-item scale [37], higher scores represent greater mastery. General self-rated health was assessed with the item "*in general, would you say your health is *(options: excellent, very good, good, fair, poor)". Self-rated physical and mental health were assessed using the RAND-12 Physical and Mental Health Component scores derived from the SF-12 [38,39]. Each scale has a mean of 50 (SD = 10) in the general population; higher scores indicate better outcomes. The Goldberg anxiety and depression scales assessed the presence of 18 psychological symptoms in the past month; scores on each scale range from 0 to 9 [40]. We used a cut-point of seven on both scales to identify severe symptomatology.

To ensure that analyses tested correlates of cervical screening service uptake rather than health-service utilisation more generally, a measure of overall health service use was also included in all statistical models [41]. This was based on the total number of Medicare services received over the same two-year period. A three-level categorical variable (low, medium and high services use) was created by applying cut-points at the upper and lower quartiles of the distribution, corresponding to 18 and 76 Medicare services used.

### Analyses

To account for initial non-response, sample probability weights were applied to all analyses [42]. These weights were derived from Census data collected for the Canberra and Queanbeyan regions in 2001 to produce representative estimates for this population. Longitudinal weights were also applied to account for individual differences in the likelihood of attrition and consent to data linkage. The amount of missing data in the survey was relatively low (see Table 1). Missing data was imputed with the STATA multiple imputation by chained equations procedure.

**Table 1:** Characteristics of the sample and proportion of missing data for each measure

			Weighted			Unweighted
Characteristics		N	% or M	95% CI	%	% missing data
**Demographic**						
Age cohort (ref = 44-48 years, n = 1141)	64-68 years	954	47.1%	44.8-49.3	45.5%	0.00%
Had child/children (ref = no, n = 192)	yes	1902	90.1%	88.8-91.2	90.8%	0.05%
Spousal status (ref = no partner, n = 602)	partnered	1492	68.0%	65.8-70.1	71.3%	0.05%
Race (ref = Caucasian, n = 2001)	not Caucasian	94	4.7%	3.7-5.7	4.5%	0.00%
Overall Medicare service use	low	497	M = 9.0	8.5-9.5	9.1%	0.00%
	medium	1073	M = 39.8	38.7-40.8	39.6%	
	high	525	M = 141.1	134.4-147.9	139.2%	
**Socio-economic**						
Financial hardship (ref = no hardship, n = 1980)	1 or more	109	0.6%	4.9-7.3	5.2%	0.29%
Welfare receipt (ref = not main income, n = 1755)	main income	335	18.9%	17.0-20.8	16.0%	0.24%
Housing (ref = own home, n = 1885)	renting	205	11.1%	9.6-12.6	9.8%	0.24%
Employment status	working	1173	51.9%	49.7-54.2		0.10%
	unemployed	24	1.2%	0.7-1.7	1.2%	
	not in labour force	896	46.8%	44.5-49.1	42.8%	
Grew up in poverty (ref = no, n = 1836)	yes	258	13.3%	11.7-14.9	12.3%	0.05%
Highest educational attainment	< high school	629	32.8%	30.7-35.0		0.00%
	< tertiary	773	41.8%	39.5-44.1	38.9%	
	tertiary degree	692	25.3%	23.5-27.2	33.1%	
**Lifestyle risk factors**						
BMI (ref = < 30, n = 1548)	≥ 30 (obese)	455	24.1%	22.0-26.1	22.7%	4.39%
Smoking status (ref = never/former, n = 1836)	current	257	14.1%	12.4-15.8	12.3%	0.10%
Illicit drug use (ref = none, n = 1500)	former/current	590	27.6%	25.5-29.6	28.2%	0.24%
Physical activity (ref = moderate/vigorous, n = 1219)	little/none	816	41.1%	38.9-43.4	40.1%	2.86%
Alcohol use (ref = none to medium, n = 1954)	hazardous/harmful	137	6.3%	5.2-7.3	6.6%	0.19%
**Traumatic events (ref = not experienced)**						
Early sexual experience (n = 2047)		48	2.5%	1.7-3.2	2.3%	0.00%
Lifetime rape (n = 1927)		145	7.7%	6.4-9.0	7.0%	1.10%
Lifetime sexual molestation (n = 1675)		396	19.6%	17.7-21.4	19.1%	1.15%
Childhood sexual abuse (n = 2051)		43	2.1%	1.5-2.8	2.1%	0.05%
Childhood physical abuse (n = 1984)		110	5.6%	4.5-6.7	5.3%	0.05%
**Personality**						
Neuroticism		2079	M = 4.1	3.94-4.24	4.06	0.76%
Extroversion		2070	M = 6.8	6.66-6.97	6.82	1.19%
Mastery		2078	M = 21.4	21.3-21.6	21.57	0.91%
**Health**						
Hysterectomy (ref = no, n = 1685)	yes	410	20.8%	18.9-22.7	0.196	0.00%
Self-rated health (ref ≥ good, n = 1839)	fair/poor	252	13.5%	11.8-15.1	0.121	0.19%
RAND mental health		2066	M = 49.7	49.2-50.1	49.85	1.38%
RAND physical functioning		2050	M = 48.3	47.8-48.8	48.85	2.15%
Goldberg anxiety (ref = < 7, n = 1800)	≥ 7	295	15.0%	13.3-16.7	0.141	0.62%
Goldberg depression (ref = < 7, n = 1952)	≥ 7	143	7.4%	6.2-8.7	0.068	0.81%

Initially, we contrast weighted estimates of participation in cervical screening derived from the PATH sample with published administrative data. Logistic regression models were then used to evaluate the association between individual characteristics and participation in cervical cancer screening. Odds ratios and 95% confidence intervals (95% CI) are reported. A multivariate model was developed based on the significant univariate terms and using a step-wise process to remove non-significant terms to arrive at the most parsimonious model of screening participation. These exclusion decisions were confirmed using non-weighted data and testing that the log-likelihood statistic (a measure of model fit) did not significantly change between models or differ between the starting and final models. Interactions terms for age cohort and each covariate were also tested to determine whether its relationship with cervical screening participation varied as a function of age. This was the case for overall health service use only; indicating that whilst the positive association between (greater) overall service use and cervical screening was significant for both cohorts, it was stronger for the older cohort (who demonstrated lower rates of cervical screening). This confirmed our adjustment for age cohort in all presented analyses.

We used analysis of Population Attributable Risk (PAR) to estimate the proportion of women in the population whose non-screening could be attributed to readily observable risk factors. A summary PAR was calculated based on logistic regression results [43] using the STATA aflogit command to identify the reduction in non-participation in screening if inequalities were eliminated.

## Results

Table 1 analyses are based on data from 1141 women in the middle-aged cohort and 954 women in the older-aged cohort (N = 2095). The remaining analyses exclude women who report hysterectomy (n = 146 in middle-aged and n = 267 in the older cohort), resulting in a total sample of 1685 women. Table 1 reports the characteristics of the sample, presenting weighted and unweighted estimates and the proportion of missing data for each measure. It is evident that missing data is relatively rare, with only the measures of BMI, exercise and the RAND-12 physical functioning scale having over 2% missing data. The majority of women in the study have children, are married and are Caucasian. Welfare reliance and financial hardship are rare, though almost half of the sample is not participating in the workforce (reflecting the norm for the older cohort).

Table 2 contrasts the estimates of two-year cervical cancer screening derived from the PATH sample (excluding women who have had a hysterectomy) with comparable administrative data for the Australian Capital Territory. The estimates from the PATH survey are within or on the cusp of the 95% confidence interval.

**Table 2:** Comparison of estimated screening rate from PATH Project with published administrative data for the Australian Capital Territory (ACT)

Source	Age group	Percent	95% confidence interval
PATH	44-48	63.8	60.7-67.0
	64-68	55.9	51.9-59.9
	Overall	60.5	58.0-63.0
AIHW*: ACT	45-49	67.3	
	65-69	57.9	

* Australian Institute of Health and Welfare [4]

Results from analyses of the correlates of screening participation are presented in Table 3. Women in the younger cohort, those who had children, those with a partner and those with higher levels of overall Medicare service use were more likely to participate in cervical cancer screening. Within the socio-economic measures, being reliant on government welfare payments, living in rental housing and not working were associated with lower likelihood of participating. For lifestyle factors: the odds of screening participation were lower for women who were obese and current smokers, and higher for women who reported use of illicit drugs (marijuana, ecstasy or amphetamines) during their lifetime. Experience of sexual abuse in childhood was associated with lower rates of cervical cancer screening. None of the measures of personality were significantly associated with screening participation. Finally, poor self-rated health, lower levels of physical functioning and anxiety and depression symptoms were also associated with lower rates of screening.

**Table 3:** Odds ratios of cervical cancer screening by demographic, socio-economic, lifestyle, personal and health-related characteristics

		Model A Univariate		Model B Multivariate		Model C Multivariate (without service use)	
**Characteristics**		**OR**	**95% CI**	**OR**	**95% CI**	**OR**	**95% CI**
**Demographic**							
Age group	44-48	1.00			1.00		
	64-68	**0.72**			**0.44**	**0.30**-**0.64**	
Had child/children	No	1.00			1.00		1.00
	Yes	**1.64**	**1.16**-**2.31**	**1.85**	**1.26**-**2.73**	**1.67**	**1.15**-**2.41**
Spousal status	no partner	1.00					
	has partner	**1.56**	**1.24**-**1.96**				
Race	Caucasian	1.00					
	not Caucasian	0.87	0.53-1.43				
Overall Medicare use	low	1.00					
	medium	**2.86**	**2.24**-**3.67**	**4.80**	**3.57**-**6.46**		
	high	**2.28**	**1.68**-**3.09**	**7.10**	**4.62-10.92**		
**Socio-economic**							
Financial hardship	None	1.00					
	1 or more	0.61	0.38-1.00				
Welfare receipt	not main income	1.00			1.00		1.00
	main income	**0.47**	**0.35**-**0.63**	**0.65**	**0.46**-**0.92**	**0.60**	**0.43**-**0.84**
Housing	own home	1.00					
	renting	**0.64**	**0.45**-**0.91**				
Employment	unemployed/not in labour force	1.00			1.00		1.00
	working	**1.65**	**1.33**-**2.04**	**1.42**	**1.02**-**1.99**	**1.44**	**1.12**-**1.85**
Grew up in poverty	no	1.00					
	yes	0.81	0.59-1.10				
Highest educational attainment	< high-school	1.00					
	< tertiary	0.93	0.72-1.21				
	tertiary degree	1.08	0.83-1.39				
**Lifestyle risk factors**							
BMI	< 30	1.00			1.00		1.00
	≥ 30 (obese)	**0.74**	**0.57-0.96**	**0.70**	**0.52**-**0.94**	**0.76**	**0.58**-**0.99**
Smoking status	never/former	1.00			1.00		1.00
	current smoker	**0.53**	**0.39**-**0.72**	**0.51**	**0.36**-**0.74**	**0.47**	**0.34**-**0.66**
Illicit drug use	none	1.00			1.00		1.00
	former/current	**1.32**	**1.05**-**1.66**	**1.40**	**1.05**-**1.87**	**1.34**	**1.03**-**1.74**
Physical activity	moderate/vigorous	1.00					
	little/no exercise	0.82	0.66-1.02				
Alcohol	none to medium						
	hazardous/harmful	1.16	0.76-1.75				
**Traumatic events (reference = not experienced)**							
Lifetime rape		0.90	0.59-1.38				
Lifetime sexual molestation		1.08	0.82-1.41				
Childhood sexual abuse		**0.44**	**0.21**-**0.90**	**0.42**	**0.19**-**0.90**	**0.42**	**0.20**-**0.91**
Childhood physical abuse		0.65	0.41-1.04				
Early sexual experience		1.51	0.73-3.16				
Personality							
Neuroticism		1.00	0.87-1.15				
Extroversion		1.16	0.97-1.40				
Mastery		1.12	0.96-1.30				
**Health**							
Self-rated health	excellent/very good/good	1.00					
	fair/poor	**0.54**	**0.39**-**0.75**				
RAND physical functioning		**1.39**	**1.21**-**1.59**	**1.24**	**1.04**-**1.48**		
RAND mental health		1.10	0.96-1.26				
Goldberg anxiety scale	< 7	1.00			1.00		1.00
	≥ 7	**0.68**	**0.50-0.92**	**0.59**	**0.41**-**0.83**	**0.70**	**0.52**-**0.96**
Goldberg depression scale	< 7	1.00					
	≥ 7	**0.63**	**0.42**-**0.95**				

Model B in Table 3 presents the significant independent correlates of cervical cancer screening in accordance with Australian guidelines. These factors were: younger age, having had children, having a partner, higher overall use of Medicare services, not being welfare reliant, being employed, not being obese, not being a current smoker, a reported history of drug use, not reporting childhood sexual abuse, higher levels of physical functioning, and not reporting symptoms of anxiety. Odds ratios for all non-significant, univariate variables listed in Model A were also calculated with adjustment for the factors included in the final multivariate Model B model (see Additional file 1: Table S1). These variables remained non-significant following this adjustment.

Model B was replicated without adjustment for overall health service use to address concerns that this covariate may confound a model for cervical screening service use given that the former is causally related to the latter (see Model C, Table 3). The estimates for Model C are comparable to Model B, indicating that adjustment for overall service use had minimal impact on sociodemographic and personal predictors of cervical screening specifically. Two variables, age cohort and physical functioning, were no longer significantly associated with cervical screening when overall health service use was not controlled. This indicates that the associations between these two characteristics and cervical screening may only be revealed when the higher rates of overall health service use of older women and those with poorer physical functioning are accounted for. Repeating all models with unweighted and imputed data replicated the findings.

A summary PAR, based on a logistic regression model with 'not screened' as the outcome was calculated to quantify inequalities in screening associated with risk factors likely to be known or readily observed by a clinician (not having children, not being partnered, receiving income support payments, not working, obese, current smoker, anxiety, poor physical health, low health service use). The summary PAR associated with these factors was 74% (95% CI: 62.4%-81.9%). That is, 74% of non-participation in screening within national guidelines can be attributed to screening inequalities associated with the above risk factors. Put another way, the number of women *not *being screened could potentially be reduced by up to 74% if these inequalities were simultaneously and completely eliminated. The summary PAR for all factors excluding service use was 46% (95% CI: 38.5-52.2).

## Discussion

We examined the factors associated with women's participation in cervical cancer screening services in accordance with national guidelines using linked survey and administrative data. Overall, cervical screening participation rates were consistent with relevant administrative statistics, with around 60% of women having been screened over a two year period. In order of the strength of association, the independent predictors were high and moderate overall health service use, not reporting childhood sexual abuse, younger age, non-smoking, having children, no/low levels of anxiety symptoms, lifetime reported drug use, not being welfare reliant, being employed, not being obese, and high levels of physical functioning. To illustrate the implications of these findings, we used PAR to quantify the level of non-participation in screening that reflected inequalities associated with characteristics likely to be known by a woman's clinician. Approximately 75% of non-participation in cervical cancer screening within the recommended two-year period was attributable to these observable risk-factors; or 50% of non-participation if overall health service use was not accounted for. It is unrealistic to expect that, in a clinical setting, all of these demographic, socio-economic, lifestyle, trauma and health risk barriers to screening can be totally and concurrently resolved so that screening participation rates exactly aligned with the guidelines. Individual autonomy, broader life circumstances, and the multiple demands placed on clinicians are additional factors that will influence whether these risk factors can be modified and how much. However, this finding does demonstrate the potential gains that could be achieved from intervention efforts to address the identified inequalities in screening and effective targeting of health promotion messages.

### Strengths and limitations

This study addressed a significant gap in health services research on cervical (and other) screening participation by linking administrative data with extensive survey data from a community sample. This illustrates a valuable approach to investigating the personal and social determinants of a health service use, which addresses limitations evident in previous research: the fallibility of self-report data *versus *the lack of individual-level data in administrative datasets. Additionally, an overall measure of Medicare service use enabled us to control for differences in health service use more generally. The correspondence between the reference period of the administrative data and the recommended guidelines for cervical screening for Australian women supports the validity and applicability of our findings. However this study is not without shortcomings. Our overall estimate of screening participation for our sample was slightly lower than published data for the ACT but this may reflect the inclusion of regional areas of New South Wales (Queanbeyan) in the sample. Our sample was also limited to middle-aged and older women due to an absence of adequate Medicare data for the youngest cohort of PATH participants. It is possible that the reasons for cervical screening in younger women differ from these relatively older cohorts [44]. Whilst our range of predictors was more comprehensive than most previous studies, it was not exhaustive. Future research should consider age-variation in predictors of cervical screening, and the role of non-personal predictors, such as health service availability, accessibility and cost, and women's attitudes towards and knowledge about cervical cancer screening.

We are able to verify previous findings regarding the predictors of screening using reliable data. Consistent with earlier studies on cervical screening and other preventative care services, we found that markers of socio-economic disadvantage such as low household income [10] and being uninsured [11] are associated with greater odds of non-participation. This contrasts the negative association between lower socio-economic status and *greater *general health service use [41,45]. This discrepancy has been attributed to socio-economic variability in health literacy and the quality of care received by people of low and higher socio-economic backgrounds [46,47].

Also consistent with former studies were findings that higher BMI and smoking were barriers to screening as was increasing age [11]. Somewhat surprising was the finding that lifetime use of illicit drugs was associated with increased odds of screening, given that drug use would traditionally be assumed to be a marker of poor health behaviours. Previous investigation of the relationship between childhood sexual abuse and cervical screening has been equivocal with evidence for decreased screening [48] and no effect, albeit in a small sample [49]. Our finding that childhood sexual abuse was a major barrier to cervical cancer screening has important health implications given that childhood sexual abuse is also associated with high-risk for human papillomavirus [50], the causal agent for cervical cancer. The finding that higher use of Medicare services is associated with greater odds of screening may reflect the increased opportunity to be offered preventive services or the ability to access health services [41].

## Conclusions

This study illustrates a leading and reliable approach to investigating the personal and social determinants of health service use by exploiting the complementary strengths of individual survey and administrative data. Inequalities in cervical cancer screening exist despite the universality and subsidised costs of cervical cancer screening in Australia. Together with existing knowledge about factors that contribute to general health service use amongst women [51,52], the findings of this study offer an important contribution to health promotion efforts for the uptake of cervical screening services. Our results indicate that programs to improve screening uptake amongst women who are currently less likely to participate should focus on those who are not working, reliant on social welfare, currently smoke, do not have children, have poorer physical functioning, high levels of anxiety, a history of sexual abuse and low overall levels of health service use. The importance of such action is emphasised by the fact that some of these characteristics are known risk factors for the development of cervical cancer [53].

## Competing interests

The authors declare that they have no competing interests.

## Authors' contributions

SCO, PB and RJT drafted the manuscript and assisted in the design of the study. PB carried out the statistical analyses. PJ is the data manager for the PATH Study. All authors read and approved the final manuscript.

### Participant consent

Obtained

## Pre-publication history

The pre-publication history for this paper can be accessed here:

http://www.biomedcentral.com/1472-6963/12/34/prepub

## Supplementary Material

Additional file 1 **Table S1**. Odds ratios of cervical cancer screening by demographic, socioeconomic, lifestyle, personal and health-related characteristics, including non-significant factors (see Model A) adjusted for variables in the multivariate model. Click here for file

## References

[B1] Arbyn M Castellsagué X de Sanjosé S Bruni L Saraiya M Bray F Ferlay J Worldwide burden of cervical cancer in 2008 Ann Oncol 2011 22 12 2675 2686 10.1093/annonc/mdr015 21471563

[B2] Peto J Gilham C Fletcher O Matthews FE The cervical cancer epidemic that screening has prevented in the UK Lancet 2004 364 249 256 10.1016/S0140-6736(04)16674-9 15262102

[B3] Quinn M Babb P Jones J Allen E Effect of screening on incidence of and mortality from cancer of cervix in England: evaluation based on routinely collected statistics BMJ 1999 318 904 908 10.1136/bmj.318.7188.904 10102852 PMC27810

[B4] Australian Institute of Health and Welfare Cervical screening in Australia 2006-2007 Cancer Series no 47 Cat no CAN 43 2009 Canberra: AIHW

[B5] Royal Australian and New Zealand College of Obstetricians and Gynaecologists College Statement C-Gyn 5: Screening for the Prevention of Cervical Cancer 2010 Melbourne: RANZCOG http://www.ranzcog.edu.au

[B6] Centers for Disease Control and Prevention: Behavioral Risk Factor Surveillance System Women's Health 2008. Prevalence and Trend Data 2009 Atlanta: National Center for Chronic Disease Prevention and Health Promotion22338646

[B7] NHS Cancer Screening Programmes NHS Cervical Screening Programme: Annual Review 2009 2009 Sheffield: National Health Service http://www.cancerscreening.nhs.uk

[B8] Moser K Patnick J Beral V Inequalities in reported use of breast and cervical screening in Great Britain: analysis of cross sectional survey data BMJ 2009 338 doi:10.1136/bmj.b2025 10.1136/bmj.b2025 PMC2697310 19531549

[B9] Waller J Bartoszek M Marlow L Wardle J Barriers to cervical cancer screening attendance in England: a population-based survey J Med Screen 2009 16 199 204 10.1258/jms.2009.009073 20054095

[B10] Kaida A Colman I Janssen PA Recent pap tests among Canadian women: is depression a barrier to cervical cancer screening? J Women's Health 2008 17 1175 1181 10.1089/jwh.2007.0626 18699731

[B11] Nelson W Moser RP Gaffey A Waldron W Adherence to cervical cancer screening guidelines for U.S. Women aged 25-64: Data from the 2005 Health Information National Trends Survey (HINTS) J Women's Health 2009 18 1759 1768 10.1089/jwh.2009.1430 19951209 PMC2864462

[B12] Amankwah E Ngwakongnwi E Quan H Why many visible minority women in Canada do not participate in cervical cancer screening Ethn Health 2009 14 337 349 10.1080/13557850802699122 19280443

[B13] Steele CB Cardinez CJ Richardson LC Tom-Orme L Shaw KM Surveillance for health behaviors of American Indians and Alaska Natives - Findings from the behavioral risk factor surveillance system, 2000-2006 Cancer 2008 113 1131 1141 10.1002/cncr.23727 18720374

[B14] Abraido-Lanza AF Chao MT Gammon MD Breast and cervical cancer screening among Latinas and Non-Latina Whites Am J Public Health 2004 94 1393 1398 10.2105/AJPH.94.8.1393 15284049 PMC1448461

[B15] Sutton S Rutherford C Sociodemographic and attitudinal correlates of cervical screening uptake in a national sample of women in Britain Social Sci Med 2005 61 2460 2465 10.1016/j.socscimed.2005.07.017 16102881

[B16] Sabates R Feinstein L The role of education in the uptake of preventative health care: The case of cervical screening in Britain Social Sci Med 2006 62 2998 3010 10.1016/j.socscimed.2005.11.032 16403597

[B17] Coughlin SS King J Richards TB Ekwueme DU Cervical cancer screening among women in metropolitan areas of the United States by individual-level and area-based measures of socioeconomic status, 2000-2002 Cancer Epidemiol Biomark Prev 2006 15 2154 2159 10.1158/1055-9965.EPI-05-0914 17119040

[B18] Bowman JA Redman S Dickinson JA Gibberd R Sanson-Fisher RW The accuracy of Pap smear utilization self-report: a methodological consideration in cervical screening research Health Serv Res 1991 26 97 107 2016170 PMC1069812

[B19] Johnson T O'Rourke D Burris J Warnecke RB An investigation of the effects of social desirability on the validity of self-reports of cancer screening behaviors Med Care 2005 43 565 573 10.1097/01.mlr.0000163648.26493.70 15908851

[B20] Bowman JA Sanson-Fisher R Redman S The accuracy of self-reported Pap smear utilisation Soc Sci Med 1997 44 969 976 10.1016/S0277-9536(96)00222-5 9089918

[B21] Pizarro J Schneider TR Salovey P A source of error in self-reports of pap test utilization J Community Health 2002 27 351 356 10.1023/A:1019888627113 12238733

[B22] Caplan LS McQueen DV Qualters JR Leff M Garrett C Calonge N Validity of women's self-reports of cancer screening test utilization in a managed care population Cancer Epidemiol Biomarkers Prev 2003 12 1182 1187 14652278

[B23] Arbyn M Simoens C Van Oyen H Foidart J-M Goffin F Simon P Fabri V Analysis of 13 million individual patient records pertaining to Pap smears, colposcopies, biopsies and surgery on the uterine cervix (Belgium, 1996-2000) Preventive Med 2009 48 438 443 10.1016/j.ypmed.2009.02.021 19272405

[B24] Arbyn M Quataert P Hal G Oyen HV Cervical cancer screening in the Flemish region (Belgium): measurement of the attendance rate by telephone interview Eur J Cancer Prev 1997 6 389 398 10.1097/00008469-199708000-00012 9370103

[B25] Bancej CM Maxwell CJ Snider J Inconsistent self-reported mammography history: findings from the national population health survey longitudinal cohort BMC Health Serv Res 2004 4 32 10.1186/1472-6963-4-32 15541176 PMC535807

[B26] Fiscella K Holt K Meldrum S Franks P Disparities in preventive procedures: comparisons of self-report and Medicare claims data BMC Health Serv Res 2006 6 122 10.1186/1472-6963-6-122 17010195 PMC1592485

[B27] Bhandari A Wagner T Self-reported utilization of health care services: improving measurement and accuracy Med Care Res Rev 2006 63 217 235 10.1177/1077558705285298 16595412

[B28] Paskett ED McLaughlin JM Reiter PL Lehman AM Rhoda DA Katz ML Hade EM Post DM Ruffin MT Psychosocial predictors of adherence to risk-appropriate cervical cancer screening guidelines: a cross sectional study of women in Ohio Appalachia participating in the Community Awareness Resources and Education (CARE) project Prev Med 2010 50 74 80 10.1016/j.ypmed.2009.09.001 19744509 PMC2813897

[B29] Parslow RA Jorm AF Christensen H Rodgers B Use of medical services after participation in a community-based epidemiological health survey Soc Psychiatry Psychiatr Epidemiol 2004 39 311 317 10.1007/s00127-004-0747-3 15085334

[B30] Tait RJ Anstey KJ Butterworth P Incidence of self-reported brain injury and the relationship with substance abuse: findings from a longitudinal community survey BMC Pub Health 2010 10 171 10.1186/1471-2458-10-171 PMC2860359 20350327

[B31] Australian Bureau of Statistics Trewin D Household income, living standards and financial stress Year Book Australia 2002, Cat no 13010 2002 Canberra: ABS

[B32] Butterworth P Rodgers B Windsor TD Financial hardship, socio-economic position and depression: Results from the PATH Through Life Survey Soc Sci Med 2009 69 229 237 10.1016/j.socscimed.2009.05.008 19501441

[B33] Saunders JB Aasland OG Babor TF de la Fuente JR Grant M Development of the Alcohol Use Disorders Identification Test (AUDIT): WHO collaborative project on early detection of persons with harmful alcohol consumption - II Addiction 1993 88 791 804 10.1111/j.1360-0443.1993.tb02093.x 8329970

[B34] National Health and Medical Research Council Australian Alcohol Guidelines: Health Risks and Benefits 2001 Canberra: NHMRC

[B35] Stafford M Hemingway H Stansfeld SA Brunner E Marmot M Behavioural and biological correlates of physical functioning in middle aged office workers: the UK Whitehall II study J Epidemiol Community Health 1998 52 353 358 10.1136/jech.52.6.353 9764255 PMC1756727

[B36] Eysenck S Eysenck H Barrett P A revised version of the psychoticism scale Pers Individ Dif 1985 6 21 29 10.1016/0191-8869(85)90026-1

[B37] Pearlin L Lieberman M Menaghan E Mullan J The stress process J Health Soc Behav 1981 22 337 356 10.2307/2136676 7320473

[B38] Hays RD RAND-36 Health Status Inventory 1998 San Antonio: The Psychological Corporation

[B39] Ware J Kosinski M Keller S A 12-item short-form health survey: construction of scales and preliminary tests of reliability and validity Med Care 1996 34 220 233 10.1097/00005650-199603000-00003 8628042

[B40] Goldberg D Bridges K Duncan-Jones P Grayson D Detecting anxiety and depression in general medical settings BMJ 1988 297 897 899 10.1136/bmj.297.6653.897 3140969 PMC1834427

[B41] Scott TL Gazmararian JA Williams MV Baker DW Health literacy and preventive health care use among Medicare enrollees in a managed care organization Med Care 2002 40 395 404 10.1097/00005650-200205000-00005 11961474

[B42] Butterworth P Olesen S Leach L Jacomb T Calculating sample weights for the PATH Through Life survey Psychiatric Epidemiology and Social Issues Working Paper Series 2010 01 1 11 http://cmhr/working_paper.php

[B43] Bruzzi P Green SB Byar DP Brinton LA Schairer C Estimating the Population Attributable Risk for multiple risk factors using case-control data Am J Epidemiol 1985 122 904 914 4050778 10.1093/oxfordjournals.aje.a114174

[B44] Sasieni P Adams J Cuzick J Benefit of cervical screening at different ages: evidence from the UK audit of screening histories Br J Cancer 2003 89 88 93 10.1038/sj.bjc.6600974 12838306 PMC2394236

[B45] Lorant V Boland B Humblet P Deliège D Equity in prevention and health care J Epidemiol Community Health 2002 56 510 516 10.1136/jech.56.7.510 12080158 PMC1732200

[B46] Aldrich R Kemp L Williams JS Harris E Simpson S Wilson A McGill K Byles J Lowe J Jackson T Using socioeconomic evidence in clinical practice guidelines BMJ 2003 327 1283 1285 10.1136/bmj.327.7426.1283 14644976 PMC286256

[B47] Campbell SM Hann M Hacker J Burns C Oliver D Thapar A Mead N Safran DG Roland MO Identifying predictors of high quality care in English general practice: observational study BMJ 2001 323 784 10.1136/bmj.323.7316.784 11588082 PMC57358

[B48] Farley M Golding JM Minkoff JR Is a history of trauma associated with a reduced likelihood of cervical cancer screening? J Fam Pract 2002 51 827 831 12401150

[B49] Leeners B Stiller R Block E Gorres G Imthurn B Rath W Effect of childhood sexual abuse on gynecological care as an adult Psychosomatics 2002 48 385 393 10.1176/appi.psy.48.5.385 17878496

[B50] Wingood GM Seth P DiClemente RJ Robinson LS Association of sexual abuse with incident high-risk human papillomavirus infection among young African-American women Sex Transm Dis 2009 36 784 786 10.1097/OLQ.0b013e3181b3567e 19704392 PMC2787680

[B51] Parslow R Jorm A Christensen H Jacomb P Rodgers B Gender differences in factors affecting use of health services: an analysis of a community study of middle-aged and older Australians Soc Sci Med 2004 59 2121 2129 10.1016/j.socscimed.2004.03.018 15351477

[B52] Green CA Pope CR Gender, psychosocial factors and the use of medical services: a longitudinal analysis Social Sci Med 1999 48 1363 1372 10.1016/S0277-9536(98)00440-7 10369437

[B53] Dillner J Lehtinen M Bjorge T Luostarinen T Youngman L Jellum E Koskela P Gislefoss RE Hallmans G Paavonen J Prospective seroepidemiologic study of human papillomavirus infection as a risk factor for invasive cervical cancer J Natl Cancer Inst 1997 89 1293 1299 10.1093/jnci/89.17.1293 9293920

