# Supplementary material for: Personal factors influence use of cervical cancer screening services: epidemiological survey and linked administrative data address the limitations of previous research
Source: BMC Health Serv Res. 2012 Feb 14;12:34. doi: 10.1186/1472-6963-12-34 (PMC3306758; doi:10.1186/1472-6963-12-34)
Supplement: Additional file 1 — Table S1. Odds ratios of cervical cancer screening by demographic, socioeconomic, lifestyle, personal and health-related characteristics, including non-significant factors (see Model A) adjusted for variables in the multivariate model. [file 1472-6963-12-34-S1.DOC]

**Odds ratios of cervical cancer screening by demographic, socio-economic, lifestyle, personal and health-related characteristics**, **including non-significant factors (see Model A) adjusted for variables in the multivariate model.**

|  | Model A  Univariate | | Model B  Multivariate | | Model C  Multivariate  (without service use) | | Model D  Adjusted univariate* | |
| --- | --- | --- | --- | --- | --- | --- | --- | --- |
| **Characteristics** | OR | 95% CI | OR | 95% CI | OR | 95% CI | OR | 95% CI |
| **Demographic** |  |  |  |  |  |  |  |  |
| Age group 44-48 | 1 |  | 1 |  |  |  |  |  |
| 64-68 | **0.72** | **0.58 – 0.89** | **0.44** | **0.30 – 0.64** |  |  |  |  |
| Had child/children No | 1 |  | 1 |  | 1 |  |  |  |
| Yes | **1.64** | **1.16 – 2.31** | **1.85** | **1.26 – 2.73** | **1.67** | **1.15 – 2.41** |  |  |
| Spousal status no partner | 1 |  |  |  |  |  | 1 |  |
| has partner | **1.56** | **1.24 – 1.96** |  |  |  |  | 1.15 | 0.87 – 1.51 |
| Race Caucasian | 1 |  |  |  |  |  | 1 |  |
| not Caucasian | 0.87 | 0.53 – 1.43 |  |  |  |  | 0.78 | 0.43 – 1.42 |
| Overall Medicare use low | 1 |  |  |  |  |  |  |  |
| medium | **2.86** | **2.24 – 3.67** | **4.80** | **3.57 – 6.46** |  |  |  |  |
| high | **2.28** | **1.68 – 3.09** | **7.10** | **4.62 – 10.92** |  |  |  |  |
| **Socio-economic** |  |  |  |  |  |  |  |  |
| Financial hardship None | 1 |  |  |  |  |  | 1 |  |
| 1 or more | 0.61 | 0.38 – 1.00 |  |  |  |  | 1.00 | 0.57 – 1.70 |
| Welfare receipt not main income | 1 |  | 1 |  | 1 |  |  |  |
| main income | **0.47** | **0.35 – 0.63** | **0.65** | **0.46 – 0.92** | **0.60** | **0.43 – 0.84** |  |  |
| Housing own home | 1 |  |  |  |  |  | 1 |  |
| renting | **0.64** | **0.45 – 0.91** |  |  |  |  | 0.93 | 0.63 – 1.37 |
| Employment unemployed/not in labour force | 1 |  | 1 |  | 1 |  |  |  |
| working | **1.65** | **1.33 – 2.04** | **1.42** | **1.02 – 1.99** | **1.44** | **1.12 – 1.85** |  |  |
| Grew up in poverty no | 1 |  |  |  |  |  | 1 |  |
| yes | 0.81 | 0.59 – 1.10 |  |  |  |  | 0.90 | 0.63 – 1.28 |
| Highest educational attainment < high-school | 1 |  |  |  |  |  | 1 |  |
| < tertiary | 0.93 | 0.72 – 1.21 |  |  |  |  | 1.12 | 0.83 – 1.50 |
| tertiary degree | 1.08 | 0.83 – 1.39 |  |  |  |  | 0.92 | 0.69 – 1.22 |
| **Lifestyle risk factors** |  |  |  |  |  |  |  |  |
| BMI < 30 | 1 |  | 1 |  | 1 |  |  |  |
| ≥ 30 (obese) | **0.74** | **0.57 – 0.96** | **0.70** | **0.52 – 0.94** | **0.76** | **0.58 – 0.99** |  |  |
| Smoking status never/former | 1 |  | 1 |  | 1 |  |  |  |
| current smoker | **0.53** | **0.39 – 0.72** | **0.51** | **0.36 – 0.74** | **0.47** | **0.34 – 0.66** |  |  |
| Illicit drug use none | 1 |  | 1 |  | 1 |  |  |  |
| former/current | **1.32** | **1.05 – 1.66** | **1.40** | **1.05 – 1.87** | **1.34** | **1.03 – 1.74** |  |  |
| Physical activity moderate/vigorous | 1 |  |  |  |  |  | 1 |  |
| little/no exercise | 0.82 | 0.66 – 1.02 |  |  |  |  | 0.88 | 0.69 – 1.12 |
| Alcohol use none to medium |  |  |  |  |  |  | 1 |  |
| hazardous/harmful | 1.16 | 0.76 – 1.75 |  |  |  |  | 1.03 | 0.62 – 1.71 |
| **Traumatic events (reference = not experienced)** | | |  |  |  |  |  |  |
| Lifetime rape | 0.90 | 0.59 – 1.38 |  |  |  |  | 1.00 | 0.61 – 1.65 |
| Lifetime sexual molestation | 1.08 | 0.82 – 1.41 |  |  |  |  | 1.24 | 0.91 – 1.69 |
| Childhood sexual abuse | **0.44** | **0.21 – 0.90** | **0.42** | **0.19 – 0.90** | **0.42** | **0.20 – 0.91** |  |  |
| Childhood physical abuse | 0.65 | 0.41 – 1.04 |  |  |  |  | 0.73 | 0.43 – 1.24 |
| Early sexual experience | 1.51 | 0.73 – 3.16 |  |  |  |  | 2.39 | 0.99 – 5.78 |
| **Personality** |  |  |  |  |  |  |  |  |
| Neuroticism | 1.00 | 0.87 – 1.15 |  |  |  |  | 1.10 | 0.92 – 1.32 |
| Extroversion | 1.16 | 0.97 – 1.40 |  |  |  |  | 0.95 | 0.77 – 1.18 |
| Mastery | 1.12 | 0.96 – 1.30 |  |  |  |  | 0.96 | 0.80 – 1.16 |
| **Health** |  |  |  |  |  |  |  |  |
| Self-rated health excellent/very good/good | 1 |  |  |  |  |  | 1 |  |
| fair/poor | **0.54** | **0.39 – 0.75** |  |  |  |  | 0.81 | 0.50 – 1.30 |
| RAND physical functioning | **1.39** | **1.21 – 1.59** | **1.24** | **1.04 – 1.48** |  |  |  |  |
| RAND mental health | 1.10 | 0.96 – 1.26 |  |  |  |  | 0.88 | 0.72 – 1.09 |
| Goldberg anxiety scale < 7 | 1 |  | 1 |  | 1 |  |  |  |
| ≥ 7 | **0.68** | **0.50 – 0.92** | **0.59** | **0.41 – 0.83** | **0.70** | **0.52 – 0.96** |  |  |
| Goldberg depression scale < 7 | 1 |  |  |  |  |  | 1 |  |
| ≥ 7 | **0.63** | **0.42 – 0.95** |  |  |  |  | 1.10 | 0.67 – 1.83 |

*Non-significant univariate variables (see Model A), adjusted for variables in Model B.
